# Optimization of the Drum Drying Parameters and Citric Acid Level to Produce Purple Sweet Potato (*Ipomoea batatas* L.) Powder Using Response Surface Methodology

**DOI:** 10.3390/foods10061378

**Published:** 2021-06-15

**Authors:** Sri Sampath Janaka Senevirathna, Nurul Shazini Ramli, Ezzat Mohamad Azman, Nurul Hanisah Juhari, Roselina Karim

**Affiliations:** 1 Department of Food Technology, Faculty of Food Science and Technology, Universiti Putra Malaysia, Serdang 43400, Malaysia; srisampaths@gmail.com (S.S.J.S.); ezzat@upm.edu.my (E.M.A.); 2 Department of Food Science, Faculty of Food Science and Technology, Universiti Putra Malaysia, Serdang 43400, Malaysia; shazini@upm.edu.my; 3 Department of Food Service and Management, Faculty of Food Science and Technology, Universiti Putra Malaysia, Serdang 43400, Malaysia; n_hanisah@upm.edu.my

**Keywords:** purple sweet potato, response surface methodology (RSM), HPLC-MS^n^, anthocyanin, functional

## Abstract

Purple sweet potato (PSP) is a rich source of anthocyanins, but the anthocyanin content and color can be affected by the drying method and processing condition. Response surface methodology (RSM) with a Box–Behnken design (BBD) was used to investigate the effects of citric acid (CA) concentration, steam pressure (SP) and rotation speed (DS) on the physicochemical and functional properties of drum-dried purple sweet potato powder (PSPP). The anthocyanins of the PSPP were analyzed using mass spectrometry with electrospray ionization and twelve anthocyanins were identified. The results indicated that the moisture content (4.80 ± 0.17–9.97 ± 0.03%) and water activity (0.290 ± 0.004–0.47 ± 0.001) (*p* < 0.05) decreased with increasing drum temperature as well as with reduced drum rotating speed. CA had a significant (*p* < 0.05) effect on the color and total anthocyanin content (101.83 ± 2.20–124.09 ± 2.89 mg/100 g) of the PSPP. High SP and low DS negatively affected the antioxidant properties of the PSPP. DPPH value of the PSPP ranged from 20.41 ± 0.79 to 30.79 ± 1.00 μmol TE/g. The optimal parameters were achieved at 0.59% CA, 499.8 kPa SP and 3 rpm DS.

## 1. Introduction

Sweet potato, known as *Ipomoea batatas* L., is one of the most important food crops globally [1] and is the sixth most important food crop after wheat, rice, maize, potatoes and cassava. Sweet potato is the fifth most important food crop in developing countries, playing a significant role in food security, with an annual global production of more than 105 million metric tons, of which 90 million tons are produced in the Asian region [2].

There are different varieties of sweet potato of various skin and flesh colors ranging from white, yellow, orange and light purple to deep purple. Purple-fleshed sweet potatoes are more popular among researchers due to the high concentration of anthocyanins that contributes to good antioxidant and other biological activities [2,3]. It has been reported that a sweet potato tuber has the sixth highest antioxidant content among 11 roots and tubers and it is ranked 14th among 22 vegetables [4,5]. Gras et al. [6] stated that the anthocyanin content of purple sweet potato (PSP) might vary depending on the variety, and they found that it ranged from 558 to 2477 mg/100 g DM for the studied freeze-dried high-anthocyanin PSP provenances. Furthermore, Nevara et al. [7] mentioned that the anthocyanin content of drum-dried PSP powder varied from 83.72 to 121.71 mg/100 g depending on the pretreatment given prior to processing. Consumption of sweet potato is beneficial because it contains useful dietary fiber and vitamins as well as natural food antioxidants [8]. Many studies have shown that sweet potato has various potential health beneficial effects such as antioxidative [3,8,9,10], antitumor [11], antidiabetic [1], anti-inflammatory [8], anti-obesity [11], hepatoprotective [12], antimicrobial [1] and antiaging properties [11]. Therefore, it may be feasible to incorporate sweet potato derivatives into food formulations and food products to enhance their nutritional and functional values.

PSP can be used to develop colorful products for the food industry. However, due to the susceptibility of anthocyanins to changes in processing conditions, color instability is a barrier to the industrialization of anthocyanin-containing foods [9,10,12,13,14,15,16]. Therefore, color stability of PSP should be improved to promote its usage in the food industry.

Anthocyanins are unstable at high pH; the color changes from red, purple, blue and green to yellow as the pH increases from 1 to 13 [17]. Fan et al. [18] reported that PSP anthocyanins are more stable at a pH of 2 to 4 (acidic condition) than at a pH of 5 or 6 which is the natural pH of PSP. Besides, Li et al. [19] found that citric acid can be used to retain the maximum anthocyanin content and color in oven-baked blue corn cookies.

Drying is a traditional food preservation method used for extending the shelf life of foods like fruits and vegetables. Drum drying, spray drying, freeze drying, and Refractance Window Drying^®^ are some of the commercially feasible drying technologies for the production of food powders, and each has its own limitations and advantages [20]. Drum drying is one of the most energy-efficient drying techniques used to dehydrate purees and highly viscous slurries in the manufacture of powders and flakes. The main benefits of this drying method are due to the high economic usage of heat and drying rates [21]. Desobry et al. [22] reported that drum drying retained more β-carotene compared to spray and freeze drying after 15 weeks of storage; hence, drum drying is commonly used to dehydrate fruit powders [21,23,24], vegetable or tuber powders [7,25,26], cereal flour and starches [27,28] and β-carotene [22]. These drum-dried products are widely used in breakfast cereals, bakery foods, beverages and dairy products [21,23,25,26,27,28,29]. Different kinds of drum dryers are available depending on the number of drums (single, double or twin) and various feeding mechanism (roller, spray, nip and splash feeding), but the double drum dryer is still the most extensively utilized equipment in the food industry. It consists of two horizontal rotating metal cylinders, with indirect heat transfer occurring from the surface of the heated drum to the wet product while the drum is heated by steam. The drying rate of a double drum dryer is mainly affected by steam pressure and drum rotation speed [21,25,27,28].

In the food industry, optimization of processing parameters is critical for product development. Food processing operations such as drying have been successfully optimized using response surface methodology (RSM). The Box–Behnken design (BBD) is a good design for the response surface methodology because it allows for the development of sequential designs, calculation of quadratic model parameters, use of blocks, and identification of the model’s lack of fit. Therefore, BBD is widely used in the food industry to optimize the processing conditions [23,30].

However, not much work have been reported on optimizing the drum drying parameters in the presence of citric acid in an attempt to improve the physicochemical and functional properties of any food product. Therefore, the objective of this study was to investigate the effects of different levels of citric acid (CA), drum steam pressure (SP) and drum rotation speed (DS) on the physicochemical and antioxidant properties of the PSPP via RSM using the BBD.

## 2. Materials and Methods

### 2.1. Materials

Approximately 4 kg of freshly harvested PSP tubers were purchased from a local farm (Selangor, Malaysia) at the right stage of maturity (100 days after planting). Anthocyanin standards of cyanidin-3,5-O-diglucoside chloride (98%), peonidin-3-O-glucoside chloride (98%) and delphinidin chloride (96%) were obtained from Chem Faces (Wuhan, China). Folin-Ciocalteu reagent, 2,2-diphenyl-1-picrylhydrazyl (DPPH) and gallic acid (98%) were obtained from Merck Company (Darmstadt, Germany). Trolox and 2,4,6-tripyridyl-s-triazine (TPTZ) were provided by Acros Organics (Geel, Belgium). Other chemicals and solvents used were of analytical grade bought from Fisher Scientific (Leicestershire, UK).

### 2.2. Sample Preparation

The unpeeled PSP tubers (~4 kg) were washed thoroughly, weighed and cut into cubes of approximately 3 cm (length) × 3 cm (width) × 1.5 cm (height) using a stainless-steel knife to facilitate a quick and uniform heat transfer during the steaming. Then, the PSP cubes were steamed at 100 °C for 30 min in a stainless-steel steam cooker, cooled to room temperature prior to pureeing using a bowl cutter (Mainca CM-21, Spain) for 10 min at low speed. Citric acid powder (0.5 to 1.5% wet basis) was added to the puree after one minute of mashing.

### 2.3. Drum Drying Operation

The acidified PSP puree was passed through a preheated double drum dryer (R. Simon Dryers Ltd., Nottingham, England). Preliminary studies and available literature were used to set steam pressure between 300 and 500 kPa and drum rotation speed from 1 to 3 rpm. Drum-dried PSP flakes were collected, immediately packed into laminated aluminum foil bags and sealed. Then, the dried flakes were milled using a high-speed stainless-steel grinder (IKA M20, IKA Labor-Technik, Staufan, Germany) for 20 s and sieved through a 425-μm mesh screen to obtain the PSP powder, which was sealed in laminated aluminum foil bags and stored at −20 °C for further analysis.

### 2.4. Experimental Design and Data Analysis

The experiment design was generated using RSM with the three-factor BBD to investigate the effects of three independent variables, namely, of citric acid (X_1_), steam pressure (X_2_) and drum rotation speed (X_3_), on the physicochemical and functional properties of the PSPP. Table 1 displays the independent variables and respective coded and uncoded values. The polynomial regression equation used to investigate the dependent variable (*Y*) involves the main, interaction and squared effects on the response surface as shown in Equation (1):

(1) $$\begin{array}{cc} Y={\;\beta }_{0}+\beta_{1}X_{1}+ & \beta_{2}X_{2}+\beta_{3}X_{3}+\beta_{4}X_{1}X_{2}+\beta_{5}X_{1}X_{3}+\beta_{6}X_{2}X_{3}\\ & +\beta_{7}X_{1}^{2}+\beta_{8}X_{2}^{2}{}_{\;}+\beta_{9}X_{3}^{2}{}_{\;} \end{array}$$

where, *Y* is the response calculated using the models; β_0_, β_1_, β_2_, β_3_, β_4_, β_5_, β_6_, β_7_, β_8_ and β_9_ represent the regression coefficients; and X_1_, X_2_ and X_3_ are the independent variables for citric acid, steam pressure and drum speed, respectively.

### 2.5. Physicochemical Analyses of the PSPP

#### 2.5.1. Determination of Moisture Content

The moisture content of the PSPP was determined using the oven drying method [31] and expressed as a dry basis using the following formula:

(2) $$\mathrm{MC}=\frac{\left(W_{1}-W_{2}\right)}{\begin{array}{c} (W_{2} \end{array}\begin{array}{c} -W_{3}) \end{array}}\;\times \;100$$

where, W_1_ is the initial weight of the aluminum dish and the sample; W_2_ is the final weight of the aluminum dish and the sample; and W_3_ is the weight of the aluminum dish.

#### 2.5.2. Determination of Water Activity

The PSPP’s a_w_ was determined using an AquaLab water activity meter (Model CX2, Pullman, DC, USA) [24].

#### 2.5.3. Determination of Color

The color attributes of the PSPP were measured using a colorimeter (CR-410, Konica Minolta, Japan) based on the color coordinates L* (lightness), a* (redness) and b* (yellowness) at room temperature. A white tile was used to calibrate the instrument. The hue angle and chroma were calculated according to Nevara et al. [7] using the following formulas:

(3) $$\mathrm{Hue}\;\mathrm{angle}=\tan^{-1}\left(\frac{b}{a}\right)$$

(4) $$\mathrm{Chroma}=\sqrt{(a^{2}+b^{2}})$$

#### 2.5.4. Determination of the Water Solubility Index and Water Absorption Capacity

The water solubility index (WSI) and water absorption capacity (WAC) of the PSPP were evaluated following the method described by Chang et al. [32] with minor modifications. One gram of the PSPP was mixed with 10 mL distilled water in a centrifuge tube in a water bath at 37 °C for 30 min before centrifugation at 3000 g for 10 min. The resulting supernatant was dried in a laboratory oven (Memmert, Büchenbach, Germany) at 105 °C for 24 h to obtain a constant dry solid weight and the weight of the precipitate at the bottom of the centrifuge tube was recorded. The WSI and WAC were calculated using the following equations:

(5) $$\mathrm{WSI}=\frac{\;\mathrm{residual}\;\mathrm{supernatant}’s\;\mathrm{dry}\;\mathrm{weight}\;}{\mathrm{dried}\;\mathrm{powder}’s\;\mathrm{weight}}\;\times \;100$$

(6) $$\mathrm{WAC}=\frac{\mathrm{weight}\;\mathrm{of}\;\mathrm{precipitate}\;}{\mathrm{dried}\;\mathrm{powder}’s\;\mathrm{weight}\;–\;\mathrm{residual}\;\mathrm{supernatant}’s\;\mathrm{dry}\;\mathrm{weight}\;}\;\times \;100$$

#### 2.5.5. Thermal Properties

A differential scanning calorimeter (DSC) (Mettler DSC 823e, Mettler Toledo, Spain) was used to measure the thermal properties of the PSPP. About 3–5 mg powder was mixed with distilled water (1:3) and sealed in an aluminum pan. Then, the hermetically sealed pan was heated from 30 to 115 °C at a rate of 10 °C/min. An empty pan was used as a reference. Thermal properties including To (onset temperature), Tp (peak temperature), Tc (conclusion temperature) and ΔH (enthalpy change) were recorded [33].

#### 2.5.6. Determination of pH

The pH value of the PSPP was determined using a calibrated pH meter (Mettler Toledo, Switzerland). Briefly, 2 g of PSPP was mixed with 20 mL of distilled water and the electrode was placed in the solution. pH value was taken at room temperature (28 ± 2 °C).

### 2.6. Determination of the Antioxidant Activity

#### 2.6.1. Extraction of Antioxidants

The antioxidants were extracted from the PSPP as described by Yang et al. [34] with minor modifications. Briefly, 0.5 g of the PSPP sample were mixed with 15 mL of 80% methanol in a 50-mL centrifuge tube, vortexed for 30 s and placed in the dark for 2 h in a water bath fitted with a shaker at room temperature (28 ± 2 °C). Then, the tubes were centrifuged at 3000 g for 10 min and the supernatant was collected and stored in the dark at 4 °C in capped centrifuge tubes for further analysis.

#### 2.6.2. UV Absorption Spectra Scanning

The collected extract (400 µL) was mixed with 3 mL potassium chloride buffer (pH 1.0) and the absorption spectrum was scanned from 200 nm to 700 nm using a double-beam UV-1650PC spectrophotometer (Shimadzu, Kyoto, Japan).

#### 2.6.3. High-Performance Liquid Chromatography (HPLC) and Mass Spectrometry (MS)

Mass spectrometry (MS) was performed using a Thermo Scientific Q Exactive Focus mass spectrometer (Thermo Scientific, Fremont, CA, USA) equipped with an electrospray ionization source (ESI) in the positive-ion mode. The column used was a Thermo Scientific™ Hypersil GOLD™ aQ C18 column (100 × 2.1 mm i.d.; particle size, 1.9 µm) at 25 °C. The mobile phase used for the analysis comprised solvent A (acetonitrile/water/formic acid; 5: 92: 3; *v/v/v*) and solvent B (0.1% formic acid in acetonitrile). The injection volume was 5 µL and the flow rate was 0.5 mL/min. The ratio of solvent B was changed as follows: 0–20 min, 5 to 25% B; 20–26 min, 25 to 35% B; 26–28.5 min, 35 to 55% B; 28.5–32 min, 55 to 95% B; and 32–42 min, 95 to 5% B. The ions were scanned from *m/z* 150 to *m/z* 2000 at a scan resolution of 70,000. All the data were analyzed using Qual Browser, Xcalibur (Thermo Scientific, Waltham, MA, USA) [35].

For HPLC quantification of PSPP anthocyanins, the anthocyanins were acid-hydrolyzed under high temperature. About 1 g PSPP was mixed with 10 mL 6 mol/L HCl and 30 mL 80% methanol in a tube with a screw cap. The tubes were sealed tightly and heated in a water bath at 90 °C for 90 min. The tubes were immediately cooled and brought to 50 mL with 80% methanol. The samples were centrifuged at 3000 g for 15 min and the supernatant was collected. Standards of cyanidin and peonidin at 0.2 to 0.0125 mg/mL were also hydrolyzed in the same manner [36]. HPLC analysis was performed using a Waters 2695 HPLC system equipped with a Waters 2487 dual λ absorbance detector on an RP-18 LiChrospher column, 5 μm particle size, 250 × 4.6 mm (Merck, Darmstadt, Germany). The solvents used were A, 5% (*v/v*) formic acid in water, and B, 100% methanol. Separation was achieved using gradient elution from 15 to 35% (B) in the first 15 min, keeping 60% B at 30 min, and reaching 80% (B) at 40 min and 15% at 45 min. The injection volume was 20 μL and the flow rate was 1.0 mL/min [37].

#### 2.6.4. Determination of the Total Anthocyanin Content

The total anthocyanin content (TAC) was determined using the pH differential method as described by Jiang et al. [9] with some modifications. Briefly, 0.4 mL extract was mixed with 2.6 mL potassium chloride buffer (pH 1.0) and 2.6 mL sodium acetate buffer (pH 4.5) and incubated in the dark for 30 min at room temperature. The absorbance was measured at 527 nm and 700 nm using a UV-1650PC spectrophotometer (Shimadzu, Kyoto, Japan) and distilled water was used as a reference. The total monomeric anthocyanin content was expressed as cyanidin-3-glucoside equivalent using the following equation:

(7) $$\mathrm{TAC}\;\left(\mathrm{mg}/g\right)=\frac{A}{\epsilon L}\times \mathrm{MW}\;\times \mathrm{DF}\;\times \frac{V}{m}$$

where, A = (A_527_ − A_700_)_pH 1.0_ − (A_527_ − A_700_)_pH 4.5_, MW = 449.2 g/mol (molecular weight of cyanidin-3-glucoside), V = volume of the extract (mL), L = 1 cm (cell path length), DF = dilution factor, ɛ = 26,900 (molar absorption coefficient of cyanindin-3-glucoside) and m = weight of the sample (g).

#### 2.6.5. Determination of the Total Flavonoid Content

The total flavonoid content (TFC) was determined using the method of Yea et al. [38] with some modifications. One milliliter of the sample extract was mixed with 4.0 mL distilled water; then, 300 µL of 5% (*w*/*v*) NaNO_2_ and 300 µL of 10% (*w*/*v*) AlCl_3_ were added and the solution was allowed to stand for 5 min. After this, 2.0 mL of 1 M NaOH were added. The catechin solution (0–300 mg/L) was used to plot a standard curve and the absorbance was measured at 510 nm using a UV/Vis spectrophotometer (Shimadzu, Kyoto, Japan). The TFC of the PSPP was expressed in milligrams of catechin equivalent per gram (mg CE/g) of the sample.

#### 2.6.6. Determination of the Total Phenolic Content

The total phenolic content (TPC) was determined using the Folin–Ciocalteu assay with some modifications [39]. Firstly, 0.5 mL of the extract or 0–300 µg/mL of the gallic acid solution were mixed with 0.5 mL of the Folin–Ciocalteau reagent and vortexed for 10 s. After 3 min, 2.0 mL of the 7.5% (*w*/*v*) sodium carbonate solution and 2.0 mL distilled water were added and the mixture was vortexed for 10 s and left in the dark for 2 h. The absorbance was determined at 760 nm using a UV-1650PC UV/Vis spectrophotometer. The results were expressed in milligrams of gallic acid equivalent per gram (mg GAE/g) of the sample.

#### 2.6.7. Determination of DPPH Radical Scavenging Activity

The DPPH scavenging activity of the PSPP was assayed according to the procedure described by Brand-Williams et al. [40] with some modifications. Firstly, 0.1 mL of the extracted solution were mixed with 3.9 mL of the 80-μM DPPH solution, vortexed for 10 s and left in the dark for 30 min. The absorbance was measured at 517 nm using a UV-1650PC spectrophotometer and the radical scavenging activity was derived from the standard curve of the Trolox solution (0–1000 μM). The results were reported as µmol of Trolox equivalent per gram of the sample (µmol TE/g).

#### 2.6.8. Determination of Ferric Reducing Antioxidant Power

The ferric reducing antioxidant power (FRAP) was analyzed according to Benzie et al. [41] with some modifications. A freshly prepared FRAP reagent (2.7 mL) was mixed with 30 µL PSPP extract or Trolox solution (0–2000 μM). Then, the mixture was vortexed for 10 s and incubated in the dark for 30 min before reading the absorbance at 593 nm using a UV-1650PC spectrophotometer. The results were reported as µmol of Trolox equivalent per gram of the sample (µmol TE/g).

### 2.7. Statistical Analysis and Validation of the Model

The samples were analyzed in triplicate and the data obtained were analyzed using RSM with the BBD to fit the second-order polynomial equation generated using Minitab version 17.0 (Minitab Inc., State College, PA, USA). Multiple regressions were used to correlate the independent variables to the response variables and the regression coefficients of the final models were determined. The significance and quality of the fit of the model were analyzed using analysis of variance (ANOVA). The optimization of drum drying conditions was performed using the numerical multiple optimization procedure to determine the most desirable physicochemical and functional properties of the drum-dried PSPP.

A validation test was conducted to determine the adequacy of the final reduced model and provide a recommendation for the optimized variables [42]. Then, the predicted optimum drying condition was compared with the experimental value of the response. Finally, the predicted and experimental values of each response were compared by one sample *t*-test to determine the validity of the model. If there was no significant difference (*p* > 0.05) between the predicted values and the experimental data, the final reduced model was considered valid.

## 3. Results

### 3.1. UV Absorption Spectra of the PSPP

**Figure 1:**
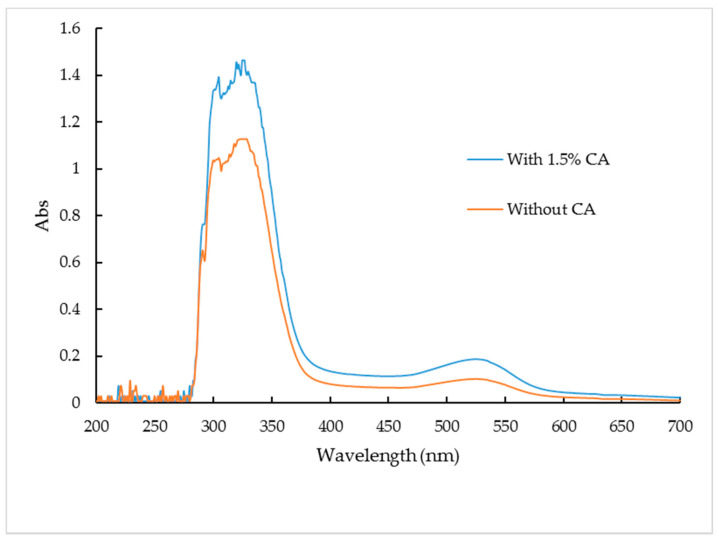
Ultraviolet-visible (UV) spectra of purple sweet potato anthocyanins. (Abs = absorbance).

The UV spectra of PSPP extracts from 200–700 nm are shown in Figure 1. Woodall et al. [43] stated that, generally, anthocyanins exhibit two absorption peaks, ranging from 270 to 290 nm and 500 to 550 nm. However, acylation of anthocyanins with aromatic organic acids gives another absorption peak in the range of 310–320 nm. In this study, four typical peaks were observed in the UV spectrum, the first peak was observed at 290–297 nm, followed by 302–306 nm and 324 and 332 nm. The final peak was observed at approximately 527 nm in agreement with the results reported by Jing Li et al. [10]. According to Jie Li et al. [12] the PSP anthocyanins are more stable at low pH value ranging from 3 to 4, under this condition the acylated anthocyanins are more stable to heat treatment, pH changes and exposure to light. Figure 1 showed that the UV spectra of the extract of drum-dried PSP added with 1.5% CA have a higher absorbance compared to the control sample, indicating that CA can be used to retain anthocyanins during drum drying of PSP.

### 3.2. Response Surface Analysis

The effects of three independent variables on moisture content (MC), water activity (aw), L*, a* and b* values, hue (Hu) and chroma (Ch), WSI, water absorption capacity (WAC), pH, TAC, TPC, TFC, radical scavenging activity (DPPH) and FRAP of the drum-dried PSPP are presented in Table 2. The independent and dependent variables were fitted to second-order polynomial as in Equation (1) to check for the goodness of fit. The result showed that the models were accurately fitted at the 95.0% confidence level, hence, can be used to predict the responses as a function of three independent variables studied (Table 2 and Table 3). All the reduced models in Table 4 had significant (*p* < 0.05) *p*-values for regression with a high R^2^ values (80.2–100%), showing the adequacy of the model fitting.

### 3.3. Model Adequacy

**Figure 2:**
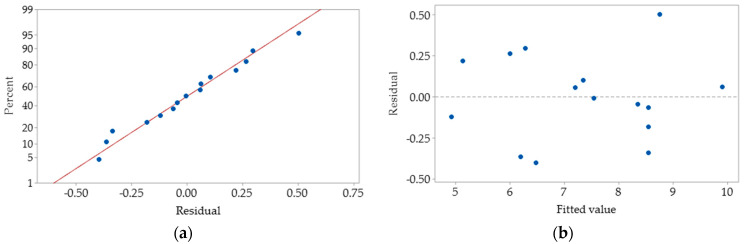
Normal probability plot (**a**) and versus fits plot (**b**) for moisture content of the PSPP (%).

The final fitted reduced models were checked for the adequacy of approximation with a real system. The adequacy of the models was judged by residuals from the least-squares fit and normal probability plots were used to check the normality assumption by constructing the residuals against normal present probability (Figure 2a). Based on the normal probability plots for all responses in this experiment, the normality assumption was fulfilled as the residual stand along a straight line in the normal probability plots [44]. In the versus fit plots, the scattered residuals on both sides of the line proved that the variances of the observations were constant for all the independent variables (Figure 2b). Based on this finding, both the normal probability plot and versus fits plot for all the responses were satisfactorily; hence, the experiential models were adequate to describe the RSM applied in this experiment.

### 3.4. Moisture Content and Water Activity

**Figure 3:**
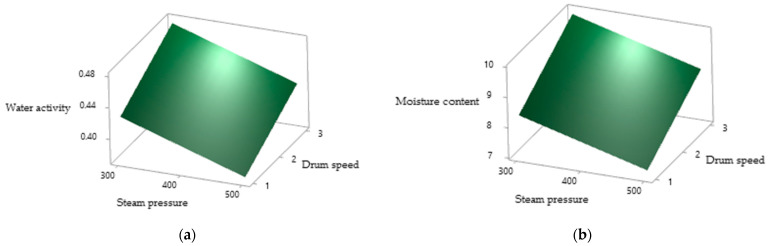
The surface plots of (**a**) water activity and (**b**) moisture content of the drum-dried PSPP as affected by the drying conditions of SP and DS.

Table 2 shows the main effect of three independent variables on MC and a_w_ and the MC of the PSPP ranged from 4.80 to 9.97%. Based on Table 4, the effect of CA and DS was significantly positive (*p* < 0.05) on MC, whereas that of SP was negative (*p* < 0.05). The DS and SP had significant positive and negative effects, respectively, on a_w_, ranging from 0.290 to 0.473. The quadratic effect of CA was negatively related to both MC and a_w,_ whereas all the other quadratic and interaction effects had an insignificant effect (*p* > 0.05) on MC and a_w_. As shown in Figure 3, the water activity and moisture content of the PSPP decreased rapidly with the increase in steam pressure, followed by a decrement in drum speed.

Pua et al. [21] also reported the comparable effect of drum drying parameters on a_w_ and MC of the drum-dried jackfruit powder. In this study, reduction of water activity and moisture content of the PSPP with the increase in steam pressure at 300, 400 and 500 kPa was due to an increase of the drum surface temperature to 104.67 ± 2.05, 114.67 ± 2.05 and 128.00 ± 1.63 °C, respectively. Similarly, Kakade et al. [26] highlighted that increasing steam pressure amplifies the drum surface temperature and increases moisture evaporation from the feed material and, hence, reduces the product’s MC. Valous et al. [28] reported a similar effect on the MC of drum-dried pregelatinized maize starches. Kakade et al. [26] further explained that high DS reduces the retention time of feed on the heated drum surface which increases the MC of the final product. Dao [25] also reported that the moisture content of the drum-dried pumpkin powder decreased with the reduction of DS but conversely increased with steam pressure.

### 3.5. Color

Table 2 summarizes the effect of CA, SP and DS on different color attributes (L*, a*, b*, hue and chroma) of the PSPP. The changes in SP and DS had no significant (*p* > 0.05) effect on the L*, b* and hue values, but had a significant (*p* < 0.05) effect on CA. The addition of CA had a significant positive effect on the a*, b*, hue and chroma values and a significant (*p* < 0.05) negative effect on L* (Table 4). Fan et al. [18] found a significant effect of pH on the hue and chroma values in their study on the color stability of anthocyanins in fermented purple sweet potatoes, which was in agreement with our findings.

Concurrently, DS had a significant positive effect on the a* and chroma values. Valous et al. [28] stated that DS was negatively related to the drum’s surface temperature at increasing DS. The destruction by heat could be minimized if the retention time of PSP puree on a heated drum surface was shorter, hence it would intensify the a* and chroma values. The quadratic effect of CA had a significant positive relationship with L* but a significant negative relationship with the a*, b*, hue and chroma values. On the other hand, the quadratic effect of SP and DS showed a significant (*p* < 0.05) positive relationship with the L* value of the drum-dried PSPP. Pua et al. [21] also reported that there was no significant effect of SP on the L* and a* values of the drum-dried jackfruit powder.

**Figure 4:**
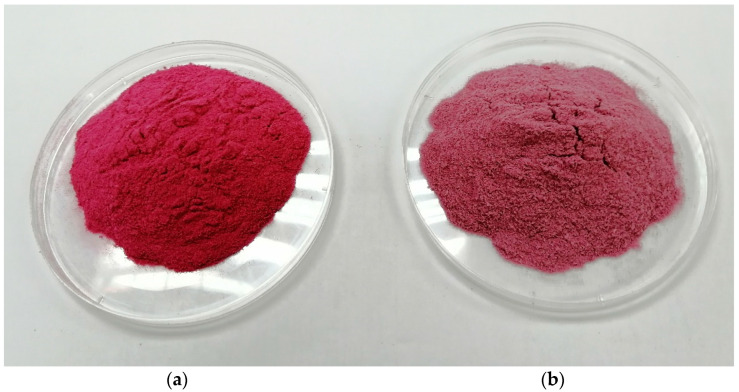
Drum-dried PSSP (**a**) with 0.6% CA and (**b**) without CA.

Color is one of the most important attributes of food that attract consumers towards buying a particular food product. The pH-dependent molecular structure of anthocyanins results in various natural colors due to structural changes in the flavylium cation, colorless carbinol pseudobases, quinoidal bases, and yellow chalcone with pH changes affecting the color stability of anthocyanins [18]. According to He et al. [13], PSP anthocyanins are more stable at low pH as they predominantly exist as flavylium cations. Acylated peonidin and cyanidins are the predominant anthocyanins in PSP and these acylated structures have a beneficial effect on color stability in acidic conditions, possibly accounting for a more intense reddish-to-pink color of the PSPP when the CA level increases. However, there was a slight color change of PSPP when SP and DS were varied compared to CA indicating that processing variables during drum drying could reduce the degradation of anthocyanins and intensify red color formation which may mask color changes due to Maillard browning reactions and caramelization [25,45]. Figure 4a,b show the color improvement of the drum-dried PSPP with and without CA under optimum SP and DS. There was a significant (*p* < 0.05) strong negative correlation (r = −0.980) between chroma and pH of the PSPP.

### 3.6. Total Anthocyanin Content

**Figure 5:**
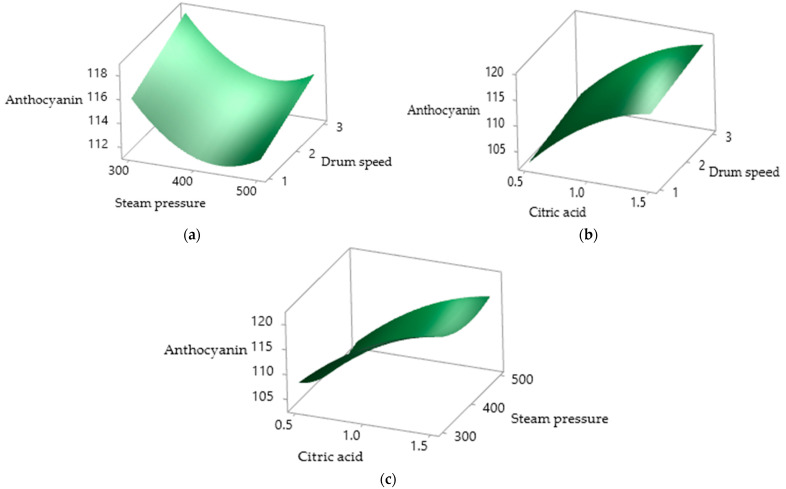
Surface plots of drum-dried PSPP anthocyanins as affected by (**a**) SP and DS; (**b**) CA and DS; (**c**) CA and SP.

CA had a positive effect on the total anthocyanin content (Figure 5), whereas SP had a significant (*p* < 0.05) negative effect (Table 3 and Table 4), ranging from 101.82 to 124.09 mg/100 g (Table 2). Both quadratic effects of CA and SP had significant (*p* < 0.05) negative and positive effects on the TAC, respectively, whereas DS had no significant (*p* > 0.05) effect on the total anthocyanin content. As shown in Table 3, the reduced model of the TAC illustrated that the increase in CA had a more positive significant effect on the TAC than other drum drying parameters. This may be due to the acylated structure of anthocyanin under acidic conditions whereby the predominant protonated flavylium cations are more stable to heat and light as a result of intramolecular co-pigmentation. The presence of a more stable pigmented complex of PSP anthocyanins may lead to positive results with a higher CA content [13,46]. Jie Li et al. [12] reported that pH significantly influenced the stability of PSP anthocyanins and a higher anthocyanin content was detected at pH 2 and 3 than at pH 5 and 6. Similarly, Fan et al. [18] discovered that PSP anthocyanins are more stable at pH 2–4 than at the natural pH of PSP i.e., pH 5–6. There was a significant (*p* < 0.05) strong negative correlation (r = −0.903) between the TAC and pH of the PSPP in the presence of CA. This could be the plausible explanation as to why CA had a positive relationship with the TAC.

Steam pressure had a significant negative effect on the TAC due to the higher surface temperature of the drum which degraded the heat-labial anthocyanins present in PSP [47]. This observation is similar to that of Durge et al. [48] who reported that retention of anthocyanins decreased with increase in the temperature of in rice extrudates, emphasizing the susceptibility of anthocyanins to high temperature. These findings were also supported by Hou et al. [49] in their study on black rice anthocyanins, whereby the degradation rate of the total anthocyanin in black rice increased at higher pH and heating temperature.

### 3.7. Antioxidant Activity

Table 2 shows the effect of CA, SP and DS on the DPPH, FRAP, TPC and TFC of the drum-dried PSPP. The DPPH of the drum-dried PSPP varied from 20.405 to 30.787 µmol TE/g, and a significant (*p* < 0.05) positive effect of DS and quadratic effect of CA on DPPH was observed. There was a significant (*p* < 0.05) negative value for the quadratic effect of SP and interaction of CA with SP. Meanwhile, CA and SP showed no significant (*p* > 0.05) effect on DPPH of the drum-dried PSPP. Jing Li et al. [10] reported that the thermal degradation of DPPH radical scavenging activity of PSP was not extreme; with only a 5.0% loss in radical scavenging activity observed in PSP anthocyanins in the citric acid–sodium citric buffer after 48 h at 90 °C.

The FRAP values observed in this study varied from 41.500 to 61.998 µmol TE/g. There was a significant (*p* < 0.05) positive effect of DS and quadratic effect of CA on the FRAP. The DPPH and FRAP increased with the increased in drum rotation speed, possibly due to the reduction in retention time of the feed on the heated drum surface, thereby causing less degradation of heat-labile anthocyanins and phenolic compounds in PSP. Furthermore, a strong positive (r = 0.838) and significant (*p* < 0.05) correlation observed between DPPH and FRAP, suggested that the scavenging activity and reducing power of the PSPP were positively related to each other.

**Figure 6:**
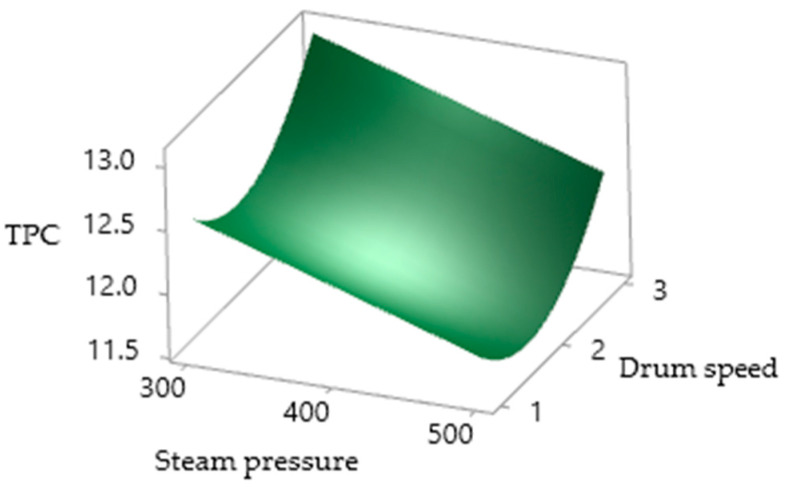
Surface plots of the drum-dried PSPP, TPC as affected by SP and DS.

There are many studies regarding the potential health benefits of phenolic compounds [50]. The TPC of the drum-dried PSPP ranged from 11.800 to 14.635 mg GAE/g and a significant (*p* < 0.05) negative effect of SP and interaction of CA with SP was detected. Meanwhile, the DS and the quadratic effect of CA and DS were significantly positive on the TPC of the PSSP. The reduction in the TPC with increasing steam pressure (Figure 6) was due to the high temperature of the drum surface leading to damage of some heat-labile bioactive and other phenolic compounds. The increase of the TPC with high DS was due to the reduced retention time of the feed on the heated drum surface. Xu et al. [50] reported that increasing temperature is more critical to the heat- labile phenolics because higher thermal energy influences the stability of bioactive compounds.

Furthermore, the TPC had a significant (*p* < 0.05) positive correlation with the DPPH (r = 0.666), FRAP (r = 0.760) and TFC (r = 0.701), illustrating that scavenging activity, power reduction and flavonoids are positively related to each other with phenolic compounds in this research.

Phenolic compounds, anthocyanins and β-carotene are mainly responsible for the antioxidant activity of foods; hence, the DPPH radical scavenging activity of PSP is mainly due to anthocyanins and other phenols [45]. Even though the antioxidant activity of food may be affected by the amount of anthocyanins and phenolic compounds, there was no correlation (*p* > 0.05) between some independent variables in this study. Shih et al. [45] also found no correlation (*p* > 0.05) between the response variables of the antioxidant capacity and other physicochemical properties of sweet potatoes.

### 3.8. Water Solubility Index and Water Absorption Capacity

Table 2 shows the effects of CA, SP and DS on the WSI and WAC of the drum-dried PSPP. The WSI of the drum-dried PSPP varied from 27.425 to 30.927%, with the WAC ranging between 663.252 and 752.04. There was a significant (*p* < 0.05) positive effect of CA and DS on the WSI, but the quadratic effect of CA and SP was found to be in significant.

**Figure 7:**
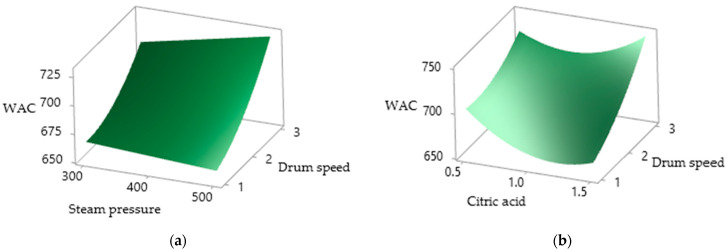
Surface plots of the drum-dried PSPP, WAC as affected by (**a**) SP and DS; (**b**) CA and DS.

Figure 7 depicts the effect of independent variables on the WAC of the PSPP, which was positively and significantly (*p* < 0.05) affected by DS, the quadratic effect of CA as well as DS and the interaction effect of CA with DS. Shih et al. [45] stated that WSI is related to the degradation of starch during processing and, according to Supprung et al. [27], this is due to the shear forces of the drum and heat causing a reduction in the water absorption capacity of starch. The positive relationship between the WAC and DS observed in this study could possibly be due to the reduced retention time of the feed on the heated drum surface. The WSI was found to be negatively correlated to pH as the addition of CA reduced the pH of the PSPP. Moreover, starch decomposition may increase under acidic conditions, leading to an increase in the WSI with the increase in CA.

### 3.9. Optimisation of Drum Drying Process Parameters for Production of PSPP

Multiple numerical optimization plots were drawn and the data are summarized in Table 5 to determine the optimum processing conditions. For overall desirability (0.8181), the concentration of citric acid, the drum drying variables of steam pressure and drum rotation speed were determined as 0.59%, 499.8 kPa and 3 rpm, respectively. These optimum conditions were predicted to lower the MC (6.791% db) and maximize the chroma (39.487), a* (39.190), TAC (108.005 mg/100g), DPPH (27.53 µmol TE/g), FRAP (56.319 µmol TE/g), TPC (13.858 mg GAE/g), pH (4.144) and WAC (736.396%).

Drum drying is a heat and mass transfer process which involves high energy utilization and the conditions can be optimized to provide an acceptable high-quality product [21]. In this study, the suggested optimized values for the drum dryer for production of an acceptable high-quality PSPP are high steam pressure and moderate-high drum rotation speed of 499.8 kPa and 3 rpm, respectively.

### 3.10. Verification of the Final Reduced Models

Validation of the final reduced model was performed by running the drum dryer under the optimum conditions and analyzing the resultant PSPP’s physicochemical and functional properties. At the optimum conditions, the predicted values for responses as shown in Table 5 were compared with the observed experimental values of MC (6.811 ± 0.147% db), chroma (39.260 ± 0.633), a* (39.247 ± 0.634), TAC (108.770 ± 1.128 mg/100 g), DPPH (27.347 ± 0.190 µmol TE/g), FRAP (55.597 ± 0.998 µmol TE/g), TPC (13.956 ± 0.224 mg GAE/g), pH (4.147 ± 0.006) and WAC (733.47 ± 7.42%). There were no significant differences (*p* > 0.05) between the predicted and experimental values, confirming the suitability of the final reduced models to optimize drum drying conditions for the PSPP.

### 3.11. HPLC-MS^n^ Analyses of Anthocyanins

The anthocyanin components of the PSPP were identified by mass spectrometry. Anthocyanins based on peonidin and cyanidin were the main components found in the PSPP (Table 6). Twelve anthocyanins were detected: cyanidin 3-sophoroside-5-glucoside, peonidin 3-sophoroside-5-glucoside, cyanidin 3-p-hydroxybenzoylsophoroside-5- glucoside, peonidin 3-p-hydroxybenzoylsophoroside-5-glucoside, cyanidin 3-(6”-feruloylsophoroside)-5-glucoside, peonidin 3-(6”-feruloylsophoroside)-5-glucoside, cyanidin 3-(6”-caffeoyl sophoroside)-5-glucoside, peonidin 3-caffeoyl sophoroside-5-glucoside, cyanidin 3-(6”,6” ‘-dicaffeoyl sophoroside)-5-glucoside, cyanidin 3-(6”-caffeoyl-6” ‘-feruloylsophoroside)-5-glucoside, peonidin 3-caffeoyl-p-hydroxybenzoyl sophoroside-5-glucoside, peonidin 3-(6”-caffeoyl-6” ‘-feruloylsophoroside)-5-glucoside.

### 3.12. HPLC Quantification of PSPP Anthocyanins

**Figure 8:**
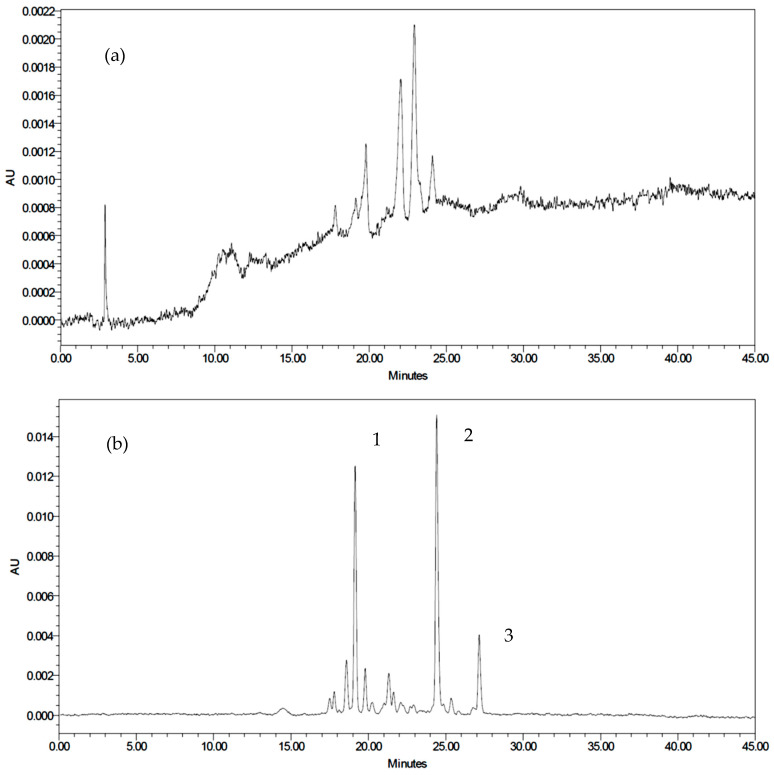
Typical (**a**) anthocyanin and (**b**) anthocyanidin HPLC profiles of the PSPP.

Since authentic anthocyanin standards were unavailable, the anthocyanin content of the PSPP was calculated as cyanidin and peonidin after acid hydrolysis of the PSPP and the peaks were compared with standard samples. Based on the retention time of the standards, peaks 2 and 3 were identified as cyanidin and peonidin, respectively (Figure 8b). Huang et al. [36] suggested that the elution order of the six major anthocyanidins should be as follows: delphinidin, cyanidin, petunidin, pelargonidin, peonidin and malvidin. However, the sample retention time of peak 1 (Figure 8b) did not match with the standard delphinidin, which confirmed that peak 1 did not belong to the six major anthocyanins. Furthermore, Fan et al. [18] reported that PSP anthocyanins mainly comprised of mono- or diacylated forms of peonidin and cyanidin. In this study, it was found that the anthocyanin content obtained by HPLC was higher than the value obtained using the pH differential method (Table 7); a similar trend was observed by Kang et al. [51] and Lee et al. [52]. Further studies are needed to determine the anthocyanidins content of the PSPP.

### 3.13. Thermal Properties of the Optimized PSP Powder

The impact of optimum drum drying conditions on the thermal properties of the PSPP was analyzed. The results showed that there was no endothermic peak observed in the DSC analysis. This finding confirmed that the drum-dried PSP starch is in an amorphous state, hence the optimum processing conditions resulted the production of an instant (pregelatinized) PSPP [53].

## 4. Conclusions

The drum drying conditions for the production of the PSPP were optimized using RSM with the three-factor BBD, namely, citric acid level (0.5–1.5%), steam pressure (300–500 kPa) and drum rotation speed (1–3 rpm). The addition of citric acid positively influenced the color parameters and TAC of the PSPP, whereas the steam pressure and drum rotation speed significantly affected the MC and antioxidant properties, with high steam pressure and low drum rotation speed providing low a_w_ and MC. The optimal processing condition for production of PSPP was achieved with steam pressure of 499.8 kPa and drum rotation speed of 3 rpm and addition of 0.59% citric acid at the overall desirability value of 0.8181 with the acceptable level of physicochemical and functional properties.

## Figures and Tables

**Table 1:** Coded and uncoded values of the independent variables.

Independent Variable	Units	Symbol	Coded and Uncoded Levels		
			−1	0	1
Citric acid (CA)	**%**	X_1_	0.5	1.0	1.5
Steam pressure (SP)	kPa	X_2_	300	400	500
Drum rotation speed (DS)	rpm	X_3_	1	2	3

**Table 2:** Box–Behnken design of RSM and the experimental data obtained using the dependent variables.

Run No.	Independent Variables			Response Variables														
	CA (%)	SP (kPa)	DS (rpm)	MC	a_w_	L*	a*	b*	Hue	Chroma	TAC	DPPH	FRAP	TPC	TFC	WSI	WAC	pH
				(%)							(mg/100 g)	(μmol TE/g)	(μmol TE/g)	(mg GAE/g)	(mg CE/g)	(%)	(%)	
1	−1	−1	0	6.57	0.387	52.27	37.33	−0.39	−0.60	37.34	107.743	23.120	49.287	13.539	4.478	27.42	704.28	4.24
2	+1	−1	0	7.45	0.391	50.10	44.47	5.58	7.15	44.82	124.089	25.175	52.229	14.336	4.683	29.03	693.50	3.50
3	−1	1	0	5.34	0.332	51.42	37.99	−0.93	−1.40	38.00	104.939	25.916	50.957	13.588	4.707	27.71	706.83	4.24
4	+1	1	0	5.83	0.290	51.27	44.44	5.03	6.46	44.72	117.145	22.670	52.682	12.808	4.587	28.38	695.01	3.50
5	−1	0	−1	4.80	0.306	50.11	36.62	−0.45	−0.71	36.62	101.825	24.934	49.038	14.336	4.719	27.74	702.49	4.25
6	+1	0	−1	6.26	0.316	48.95	44.11	5.84	7.54	44.49	116.382	23.747	49.762	13.588	4.465	29.79	665.47	3.50
7	−1	0	+1	6.07	0.321	51.61	38.23	−1.39	−2.09	38.26	105.661	28.973	61.998	14.635	4.265	28.98	734.52	4.25
8	+1	0	+1	7.54	0.366	50.21	44.67	5.54	7.07	45.01	116.625	30.787	60.855	14.252	4.410	30.29	752.05	3.51
9	0	−1	−1	8.31	0.417	48.31	42.00	4.08	5.55	42.20	115.997	20.506	46.529	12.400	4.142	28.94	664.68	3.78
10	0	+1	−1	7.26	0.374	48.20	41.52	4.34	5.97	41.75	110.971	20.405	41.500	11.800	4.052	29.07	663.25	3.78
11	0	−1	+1	9.97	0.473	47.31	43.33	4.61	6.08	43.57	116.194	23.371	47.519	13.048	4.011	29.83	698.76	3.78
12	0	+1	+1	9.25	0.462	48.57	42.82	4.52	6.02	43.05	117.236	25.496	51.326	12.180	4.050	30.93	727.66	3.78
13 ^a^	0	0	0	8.49	0.420	45.89	42.22	4.88	6.59	42.50	113.492	23.243	45.791	11.926	3.945	30.12	681.06	3.78
14 ^a^	0	0	0	8.21	0.418	45.26	42.15	5.04	6.82	42.45	114.199	24.622	44.029	12.200	4.230	29.76	685.32	3.78
15 ^a^	0	0	0	8.37	0.433	45.39	41.91	5.17	7.04	42.23	113.222	23.659	44.697	12.187	4.191	29.84	679.59	3.78

^a^ Center point; citric acid (CA) concentration, steam pressure (SP) and rotation speed (DS) of the drum drier. Moisture content (MC), water solubility index (WSI), water absorption capacity (WAC), total anthocyanin content (TAC), total phenolic content (TPC), total flavonoid content (TFC), radical scavenging activity (DPPH) and ferric reducing antioxidant power (FRAP) of the purple sweet potato powder (PSPP).

**Table 3:** Adequacy of the models fitted for the purple sweet potato powder.

Parameters	Fitted Models	R^2^	*p*-Value (Regression)	*p*-Value (Lack of Fit)
MC	MC = −1.048 + 19.63 CA − 0.0057 SP + 0.776 DS − 9.278 CA × CA	96.96	0.000	0.153
a_w_	a_w_ = 0.117 + 0.72 CA − 0.0003 SP + 0.0262 DS − 0.3579 CA × CA	88.13	0.000	0.089
L*	L = 93.350 − 32.72 CA − 0.1434 SP − 2.81 DS + 15.75 CA × CA + 0.0002 SP × SP + 0.770 DS × DS	95.55	0.000	0.186
a*	a = 29.195 + 17.24 CA − 0.0004 SP + 0.600 DS − 5.184 CA × CA	99.01	0.000	0.185
b*	b = −10.280 + 24.78 CA − 0.0011 SP − 0.068 DS − 9.247 CA × CA	98.25	0.000	0.096
Hue	Hu = −14.550 + 35.21 CA − 0.0014 SP − 0.160 DS − 13.48 CA × CA	98.16	0.000	0.114
Chroma	Ch = 28.801 + 18.24 CA − 0.0005 SP + 0.604 DS − 5.516 CA × CA	99.13	0.000	0.156
TAC	TAC = 133.500 + 33.47 CA − 0.2157 SP + 1.318 DS− 9.98 CA × CA + 0.0002 SP × SP	94.46	0.000	0.065
DPPH	DPPH = −17.430 − 9.26 CA + 0.2055 SP + 2.379 DS + 9.86 CA × CA − 0.0002 SP × SP − 0.0265 CA × SP	91.87	0.000	0.312
FRAP	FRAP = 76.910 − 60.2 CA + 0.0011 SP + 7.99 DS + 30.63 CA × CA + 3.09 DS × DS	88.10	0.000	0.100
TPC	TPC = 18.600 − 10.48 CA + 0.0042 SP − 1.585 DS + 6.677 CA × CA + 0.458 DS × DS − 0.0079 CA × SP	97.05	0.000	0.317
TFC	TFC = 6.017 − 3.61 CA + 0.0001 SP − 0.0806 DS + 1.802 CA × CA	80.59	0.000	0.675
WSI	WSI = 11.800 + 10.72 CA + 0.0548 SP + 0.561 DS − 4.657 CA × CA − 0.00006 SP × SP	87.15	0.000	0.123
WAC	WAC = 803.600 − 239.10 CA + 0.0394 SP − 41.9 DS + 87.0 CA × CA + 10.43 DS × DS + 27.27 CA × DS	95.60	0.000	0.122
pH	pH = 4.912 − 1.51 CA + 0.00001 SP − 0.0146 DS + 0.3846 CA × CA + 0.0036 DS × DS	100.0	0.000	0.393

Citric acid (CA) concentration, steam pressure (SP) and rotation speed (DS) of the drum drier. Moisture content (MC), water solubility index (WSI), water absorption capacity (WAC), total anthocyanin content (TAC), total phenolic content (TPC), total flavonoid content (TFC), radical scavenging activity (DPPH) and ferric reducing antioxidant power (FRAP) of the purple sweet potato powder (PSPP).

**Table 4:** The *p*-value and regression coefficient of the main, quadratic and interaction effect of different variables in the final reduced models fitted for purple sweet potato powder.

Response		Regression Parameter Coefficient								
		Main Effects			Quadratic Effects			Interaction Effects		
		x_1_	x_2_	x_3_	x_1_^2^	x_2_^2^	x_3_^2^	x_1_ x_2_	x_1_ x_3_	x_2_ x_3_
MC	*p*-value	0.001	0.000	0.000	0.000	-	-	-	-	-
	Coef	0.536	−0.575	0.776	−2.319	-	-	-	-	-
a_w_	*p*-value	0.803	0.011	0.010	0.000	-	-	-	-	-
	Coef	0.002	−0.026	0.026	−0.088	-	-	-	-	-
L*	*p*-value	0.027	0.436	0.274	0.000	0.001	0.049	-	-	-
	Coef	−0.611	0.185	0.265	3.937	1.816	0.770	-	-	-
a*	*p*-value	0.000	0.707	0.000	0.000	-	-	-	-	-
	Coef	3.438	−0.044	0.600	−1.296	-	-	-	-	-
b*	*p*-value	0.000	0.455	0.659	0.000	-	-	-	-	-
	Coef	3.145	−0.115	−0.067	−2.312	-	-	-	-	-
Hue	*p*-value	0.000	0.506	0.453	0.000	-	-	-	-	-
	Coef	4.128	−0.141	−0.160	−3.369	-	-	-	-	-
Chroma	*p*-value	0.000	0.671	0.000	0.000	-	-	-	-	-
	Coef	3.603	−0.049	0.604	−1.379	-	-	-	-	-
Antho	*p*-value	0.000	0.020	0.058	0.021	0.021	-	-	-	-
	Coef	6.759	−1.716	1.318	−2.494	2.482	-	-	-	-
DPPH	*p*-value	0.852	0.452	0.000	0.002	0.003	-	0.034	-	-
	Coef	−0.071	0.289	2.379	2.465	−2.201	-	−1.325	-	-
FRAP	*p*-value	0.625	0.917	0.002	0.001	-	-	-	-	-
	Coef	0.530	0.110	4.360	7.440	-	-	-	-	-
TPC	*p*-value	0.116	0.002	0.014	0.000	-	0.004	0.008	-	-
	Coef	−0.139	−0.368	0.249	1.669	-	0.458	−0.394	-	-
TFC	*p*-value	0.953	0.841	0.134	0.000	-	-	-	-	-
	Coef	−0.003	0.010	−0.081	0.451	-	-	-	-	-
WSI	*p*-value	0.002	0.523	0.007	0.001	0.020	-	-	-	-
	Coef	0.704	0.108	0.561	−1.164	−0.672	-	-	-	-
WAC	*p*-value	0.073	0.160	0.000	0.000	-	0.024	-	0.005	-
	Coef	−5.260	3.940	27.140	21.750	-	10.430	-	13.640	-
pH	*p*-value	0.000	0.189	1.000	0.000	-	0.020	-	-	-
	Coef	−0.370	0.001	0.000	0.096	-	0.004	-	-	-

Regression coefficient (Coef); x_1_, x_2_, x_3_ are dependent variables; moisture content (MC), water solubility index (WSI), water absorption capacity (WAC), total anthocyanin content (TAC), total phenolic content (TPC), total flavonoid content (TFC), radical scavenging activity (DPPH) and ferric reducing antioxidant power (FRAP) of the purple sweet potato powder (PSPP).

**Table 5:** Optimum drum drying parameters and responses for the PSSP.

Process Parameters	Target	Experimental Range		Optimum Value	Desirability
		Min	Max		
Citric acid (%)	range	0.5	1.5	0.59	
Steam pressure (kPa)	range	300	500	499.8	
Drum rotation speed (rpm)	range	1	3	3	
	Responses			Predicted values	0.8181
MC (%)	minimize	4.801	9.965	6.791	
Ch	target	36.619	45.009	39.487	
a*	target	36.617	44.667	39.190	
TAC (mg/100 g)	target	101.825	124.089	108.005	
DPPH (µmol TE/g)	maximize	20.405	30.787	27.530	
FRAP (µmol TE/g)	maximize	41.500	61.998	56.319	
TPC (mg GAE/g)	maximize	11.800	14.635	13.858	
WAC (%)	maximize	663.252	752.04	736.396	
pH	maximize	3.503	4.250	4.144	

Moisture content (MC), chroma (Ch), total anthocyanin content (TAC), total phenolic content (TPC), total flavonoid content (TFC), radical scavenging activity (DPPH) and ferric reducing antioxidant power (FRAP), water absorption capacity (WAC) of the purple sweet potato powder (PSPP).

**Table 6:** Identification of PSPP anthocyanins by mass spectrometry.

Peak	Retention Time (min)	*m/z*			Anthocyanin
		MH^+^	Aglycon	Other Fragment Ions	
1	11.63	773	287	611, 449	Cyanidin 3-sophoroside-5-glucoside
2	12.99	787	301	625, 463	Peonidin 3-sophoroside-5-glucoside
3	16.08	893	287	731, 449	Cyanidin 3-*p*-hydroxybenzoylsophoroside-5-glucoside
4	17.27	907	301	745, 463	Peonidin 3-*p*-hydroxybenzoylsophoroside-5-glucoside
5	18.24	949	287	787, 449	Cyanidin 3-(6”-feruloylsophoroside)-5-glucoside
6	19.38	963	301	801, 463	Peonidin 3-(6”-feruloylsophoroside)-5-glucoside
7	20.03	935	287	773, 449	Cyanidin 3-(6”-caffeoyl sophoroside)-5-glucoside
8	20.76	949	301	787, 463	Peonidin 3-caffeoyl sophoroside-5-glucoside
9	20.81	1097	287	935, 449	Cyanidin 3-(6”,6” ‘-dicaffeoyl sophoroside)-5-glucoside
10	21.50	1111	287	949, 449	Cyanidin 3-(6”-caffeoyl-6” ‘-feruloylsophoroside)-5-glucoside
11	21.96	1069	301	907, 463	Peonidin 3-caffeoyl-*p*-hydroxybenzoyl sophoroside-5-glucoside
12	22.52	1125	301	963, 463	Peonidin 3-(6”-caffeoyl-6” ‘-feruloylsophoroside)-5-glucoside

**Table 7:** Anthocyanins in PSPP using different method of analysis.

Analysis Method	Anthocyanin Content (mg/100 g DM)
HPLC	
Cyanidin-based	85.17 ± 1.64
Peonidin-based	65.91 ± 4.07
Total anthocyanins	151.08 ± 5.69
pH differential method	
Total anthocyanins	108.77 ± 1.13

HPLC = High-Performance Liquid Chromatography.

## Data Availability

Data is contained within the article.
